# The Modern Orthopædic Unit

**Published:** 1913-11-29

**Authors:** 


### THE ORTHOPÆDIC DEPARTMENT.

### III.—The Modern Orthopædic Unit.

Following upon its recent progress came the suggestion that this branch of surgery should be whole-heartedly recognised as a legitimate speciality in hospital practice. The necessary corollary to such recognition is the creation of a special orthopædic department, or unit, in each general hospital that claims to be up to date.

Hitherto, for the most part, orthopædists have tried to win recognition for their work and claims in special hospitals. While there is much to be said in favour of such special hospitals, the fact remains that every general hospital ought, if it wishes to be up to date and complete, to have each special department properly represented within its orbit. This contention is now so generally admitted that we need not stop to discuss it here. Suffice it to say that while at some centres the special orthopædic hospital still flourishes and does excellent work, most general hospitals have now attempted to form departments for that speciality, and by such attempts have shown that they fully realise the importance of the modern development in orthopædics. What constitutes such a unit or department?

We have practical illustrations of these innovations in America and on the Continent. One of the finest of the newer departments is that in charge of Professor Lange at the new clinic in Munich; a less elaborate, but equally efficient, department is attached to the surgical clinic at Berlin, while interesting departments are found at Stockholm, Copenhagen, Philadelphia, Boston, Turin, Bologna, Vienna, and Budapest. In England, while Mr. Jones's excellent clinic at Liverpool attracts many students who go to study his particular methods, it can scarcely be said to rank on an equality with these departments abroad; in London we have the beginnings of excellent units at St. Bartholomew's Hospital, Charing Cross, and the London Hospital. Orthopædic work is, of course, done at all the hospitals, and sometimes—as at Great Ormond Street—very efficiently, but most of the surgeons work under decided difficulties. There are indications, however, that the authorities of our larger hospitals mean to do better in future. Thus Guy's has lately established a department, and similar units will no doubt be established at King's College Hospital and University. But for the present, at least, the most adequately fitted-up and most generally interesting orthopædic department in London belongs to the hospital which it constitutes —the Royal National Orthopædic Hospital in Great Portland Street, formed some years ago by the amalgamation of the three special institutions that dealt with this subject. We may conveniently take this hospital as the basis of our ideal unit—at least for purposes of comparison and discussion.

Starting from the assumption that the department shall be self-contained, as every unit ought to be, we may postulate that it shall consist of practically three divisions. These are (1) the in-patient division, or ward; (2) the out-patient division, or examining portion; and (3) the working division in which treatment is given. The term "self-contained," so far as it applies to a unit in a general hospital, does not mean that the department forms a small hospital in itself; after all the purely administrative work is general to all departments, and in several other subdivisions material economies will be effected by so grouping the unit that it is interdependent instead of wholly self-centred. But "self-contained" does imply that the department shall be able to carry on its work properly without being handicapped by a breakdown in some other department. For that reason it is necessary that it should possess, say, its own *x*-ray room, its own medical-treatment room, and its own massage department. These units, which in themselves may be claimed to be independent departments, are so vitally necessary that their germ must be found in every department which needs them, although, of course, a central department in each of these specialities suggests itself as a necessity.

With regard to the site of the orthopædic unit little need be said, for in the majority of cases the orthopædist has to take what is given him. Where the surgeon is in a position to consult with the architect in devising plans for an entirely new department, he should bear in mind that the speciality upon which he is engaged is likely, in the future, to be considerably extended. A careful study of the designs of the newer departments abroad—we may instance that at Munich and Philadelphia—will give valuable hints which should be utilised to the full. Proper orientation of the wards and treatment rooms, so as to secure the maximum of useful sunlight, must remain a primary consideration, though this is a matter which architects and surgeons very often lose sight of. We say advisedly "useful sunlight," for it is not a fact that there is no material difference between morning and afternoon sunlight so far as a hospital ward is concerned. The difference, indeed, if modern experiments on fatigue and stimulative effect count for anything, is one that should be taken into account in hospital planning in the future. So let the ward division be planned so as to secure the maximum of morning sunshine with adequate ventilation, adequate warmth, and adequate freedom from outside disturbance. For the nature of walls, ceiling, floor, furniture, see "The Making of a Modern Hospital," The Hospital, July 27, 1912, to which we refer our readers.

We now come to the ward itself. Since the average stay of patients in the ward, owing to the somewhat chronic character of the cases treated, is decidedly longer than in a purely surgical ward, a day room becomes a necessity. An ideal day room is a difficult item to plan in a ward scheme; probably the nearest approach to it is a covered balcony with glass roofing and sides, adequately warmed, and so placed that it does not interfere with the light of the ward itself, and has easy access to the sanitary annexes. A special ward for children is, we think, a necessity; the mixing of child and adult patients is to be avoided in this as in other departments as far as possible. Both the wards should be attractive. To secure an attractive appearance limit the number of beds, giving slightly more bed space to each patient than is usual in the other wards, and have good, solid ward furniture. The extra expense entailed through this is amply compensated.

The wards should be complete ward units, with proper annexes, and, above all, with a properly fitted-up dressing room. This last, in fact, should be, in miniature, a room like that attached to the out-patient division.

In connection with the in-patient or ward division will be the main operating room, a second, for minor and manipulative surgery such as can be done in the out-patient, being provided in the latter department. The main operating theatre should conform to the established type. The operations that will be carried out here are sometimes exceedingly delicate—as in nerve surgery—and only the most careful consideration of essentials will ensure success.

